# Hierarchical linear modeling of longitudinal pedigree data for genetic association analysis

**DOI:** 10.1186/1753-6561-8-S1-S82

**Published:** 2014-06-17

**Authors:** Qihua Tan, Jacob V B Hjelmborg, Mads Thomassen, Andreas Kryger Jensen, Lene Christiansen, Kaare Christensen, Jing Hua Zhao, Torben A Kruse

**Affiliations:** 1 Institute of Clinical Research, Unit of Human Genetics, University of Southern Denmark, Sdr. Boulevard 29, 5000 Odense C, Denmark; 2 Epidemiology, Biostatistics and Biodemography, Institute of Public Health, University of Southern Denmark, J. B. Winsloews Vej 9B, 5000 Odense C, Denmark; 3 MRC Epidemiology Unit and Institute of Metabolic Science, Addenbrooke's Hospital, Cambridge CB2 0QQ, UK

## Abstract

Genetic association analysis on complex phenotypes under a longitudinal design involving pedigrees encounters the problem of correlation within pedigrees, which could affect statistical assessment of the genetic effects. Approaches have been proposed to integrate kinship correlation into the mixed-effect models to explicitly model the genetic relationship. These have proved to be an efficient way of dealing with sample clustering in pedigree data. Although current algorithms implemented in popular statistical packages are useful for adjusting relatedness in the mixed modeling of genetic effects on the mean level of a phenotype, they are not sufficiently straightforward to handle the kinship correlation on the time-dependent trajectories of a phenotype. We introduce a 2-level hierarchical linear model to separately assess the genetic associations with the mean level and the rate of change of a phenotype, integrating kinship correlation in the analysis. We apply our method to the Genetic Analysis Workshop 18 genome-wide association studies data on chromosome 3 to estimate the genetic effects on systolic blood pressure measured over time in large pedigrees. Our method identifies genetic variants associated with blood pressure with estimated inflation factors of 0.99, suggesting that our modeling of random effects efficiently handles the genetic relatedness in pedigrees. Application to simulated data captures important variants specified in the simulation. Our results show that the method is useful for genetic association studies in related samples using longitudinal design.

## Background

Genetic studies show that genes influence both the mean level and the rate of change of anthropometric traits. For example, substantial genetic contributions to the rate of change have been detected for both body mass index (BMI) [1,2] and body weight [3] using longitudinal data on twins. These results provide an epidemiological basis for molecular genetic studies to identify genetic variants that affect these traits. Genome-wide association studies (GWAS) driven by high-throughput genotyping techniques enable the mapping of genes associated with changes in human traits using the longitudinal design, which has been shown to have power advantages over the cross-sectional design [4,5]. For example, in a longitudinal GWAS conducted on cardiovascular disease (CVD) risk factors, Smith et al [6] detected single-nucleotide polymorphisms (SNPs) that influence the change in multiple traits or CVD risk factors including glucose, low-density lipoprotein, triglyceride levels, body weight, and waist circumference, among others. Another very recent longitudinal GWAS on BMI identified SNPs associated with development of obesity [7].

Just as traditional genetic epidemiology studies are frequently conducted in large pedigrees because disease-causing mutations segregate within families, so the relative ease in SNP genotyping has led to genetic association analysis in large pedigrees. For example, the Long Life Family Study (https://dsgweb.wustl.edu/llfs/), supported by the National Institute on Aging, collects high-resolution genome-wide genotype data in families with longevity probands and their offspring. With support from the NIH, Genetic Analysis Workshop 18 (GAW18) provides whole genome sequencing data and longitudinal blood pressure measurements on large pedigrees. In fact, genetic analysis on longitudinal measurements from pedigrees is an important topic calling for novel statistical modeling [8,9]. Such data are characterized by the random variation between individual longitudinal trajectories arising from repeated measurement on an individual, and by the random effect between pedigrees resulting from relatedness among individuals within a pedigree, which is one of the situations of sample stratification in GWAS [10]. An efficient mixed model was introduced to deal with sample structure resulting from genetic correlation by introducing a kinship matrix to account for pairwise relatedness between individual samples [11]. Implementation of the algorithm to GWAS in correlated samples is possible via software packages such as EMMAX [11] and kinship [12]. Although useful for adjusting relatedness in the mixed modeling of genetic effects on the mean level of a phenotype, current algorithms implemented in popular statistical packages are not sufficiently straightforward for handling the kinship correlation on the time-dependent trajectories of a phenotype. This paper introduces a novel integration of the hierarchical linear model (HLM) to handle intraindividual correlation resulting from repeated longitudinal measurements and the kinship model to deal with intrapedigree relatedness with an example application to the GAW18 data.

## Methods

### The GAW18 data

#### Phenotype data

GAW18 provides blood pressure data in 20 large pedigrees (27 to 107 individuals per pedigree, mean pedigree size 69 individuals, 932 participants in total) measured longitudinally over 4 times in a period of 30 years. In total, 246 individuals have 1, 183 have 2, 309 have 3, and 194 have 4 measurements. Besides blood pressure, information concerning hypertension diagnosis (systolic blood pressure [SBP] >140 mm Hg, diastolic blood pressure [DBP] >90 mm Hg), antihypertension medicine intake, and tobacco smoking is also collected at each examination. Participants entered the study at different ages, ranging from 16 to 94 years, with a mean age of 39.6 years.

#### Genotype data

The data contains SNP genotypes on odd-numbered autosomes for individuals in the 20 pedigrees obtained using different versions of the Illumina Infinium Beadchips. Raw genotype data were processed using standard quality control procedures and cleaned for mendelian errors. This paper focuses on chromosome 3 data, which contains 65,519 SNPs, to illustrate the application of our proposed method.

#### Simulated data

GAW18 also provides simulated phenotype data for 200 replicates, each with 849 individuals from the real pedigrees. As with the real data, longitudinal blood pressure measurements were simulated for 3 time points with 3.9 years between exams 1 and 2 and 6.9 years between exams 1 and 3. The simulation included age and sex as fixed covariates and hypertension diagnosis, medication use, and tobacco smoking as time-varying covariates.

### Hierarchical linear models

The HLM is a complex form of regression analysis [13,14], referred to as random coefficients model with 2 defining features. First, the data appropriate for HLM are structured with difference levels with lower-level or level 1 units (here, blood pressure measurements over time for each individual) nested within the higher-level or level 2 units (here, genotypes at a given SNP locus across individuals). Second, the parameters of the level 1 model characterize linear relationships occurring between level 1 units (here, the blood pressure trajectory over time). These parameters can be modeled as a function of level 2 units (genotypes). Our 2-level HLM takes the form of regression models developed separately for level 1 and level 2 units. For clarity, the level 1 model for each individual can be shown as

(1) $$Y_{ij}=\beta_{0,i}+\beta_{1,i}X_{ij}+e_{ij}$$

where *Y_ij _*is SBP measured for individual *i *at age *j*; *X_ij _*is age at measurement for individual *i*; *β_0,i _*and *β_1,i _*are the intercept and slope parameters for individual *i*; *e_ij _*is a random error associated with individual *i *at age *j *that is normally distributed with *E*(*e_ij_*) = 0, *var(e_ij_) *= *σ^2^*.

In the level 2 model, the regression coefficients from the level 1 model are regressed on the level 2 group variable, here defined as the genotypes for a SNP:

(2) $$\beta_{0,i}=\gamma_{00}+\gamma_{01}G_{i}+U_{0,i}$$

(3) $$\beta_{1,i}=\gamma_{10}+\gamma_{11}G_{i}+U_{1,i}$$

In equations (2) and (3), *G_i _*is genotype for individual *i*; *γ_00 _*and *γ_10 _*are the intercepts or overall means for *β_0,i_* and *β_1,i_*; γ_01 _and γ_11 _are the regression coefficients (slopes) associating genotypes with *β_0,i _*and *β_1,i_*; *U_0i _*and *U_1i _*are the random effects for *β_0,i _*and *β_1,i_* after adjusting for genotype *G_i _*that are normally distributed with *E(U_0i_) = E(U_1i_) = *0. In our context, the most interesting parameters are *γ_01 _*and *γ_11_*, which represent genotype association with overall mean and rate of change for SBP.

### Modeling relatedness

The HLMs described so far are most appropriate for unrelated individuals from a general population. When longitudinal data are collected in pedigrees, correlation arises among the random effects for individual trajectories described above. In human pedigrees, the heritable random variation is distributed following a kinship matrix defined according to the pairwise genotypic similarity of individuals, which can be readily derived from pedigree structures or whole genome data. Explicitly modeling the pedigree structure in a mixed-effect model is shown to improve performance in GWAS by bringing the genomic control factor very close to 1 [11,15]. Here we introduce a mixed model that involves a kinship matrix to our level 2 models to handle the relatedness resulting from pedigree structures in the GAW18 data. In the mixed model, we define *G_i _*in equations (2) and (3) as a fixed-effect variable and *U_0,i _*and *U_1,i _*as random effect variables that can be decomposed into 2 parts: a heritable component (*u_0,i _*or *u_1i_*) and a nonheritable component (*ε_0,i _*or *ε_1,i_*) such that

(4) $$U_{0,i}=u_{0,i}+\varepsilon_{0,i}$$

(5) $$U_{1,i}=u_{1,i}+\varepsilon_{1,i}$$

(6) $$Var\left(u_{0}\right)=\sigma_{0,g}^{2}K,\;Var\left(u_{1}\right)=\sigma_{1,g}^{2}K$$

In equation (6), *u_0 _*is a vector representing the heritable component in the random variation in individual intercepts, *β_0,i_*; *u_1 _*is a vector representing the heritable component in the random variation in individual slopes, *β_1,i_*; $\sigma_{0,g}^{2}$ and $\sigma_{1,g}^{2}$ represent the variability in phenotype dissimilarity among genetically related individuals; *K *is a matrix of kinship coefficients calculated from pedigree structures. In the actual estimation of the level 2 models, extra fixed-effect variables, such as sex, can be included so that effects of these fixed variables can be estimated together with that for each SNP with the possibility of estimating interactions between them. The level 2 models are fitted using the *lmekin *function in the free R package, *kinship*.

## Results

### Original phenotype data

**Figure 1:**
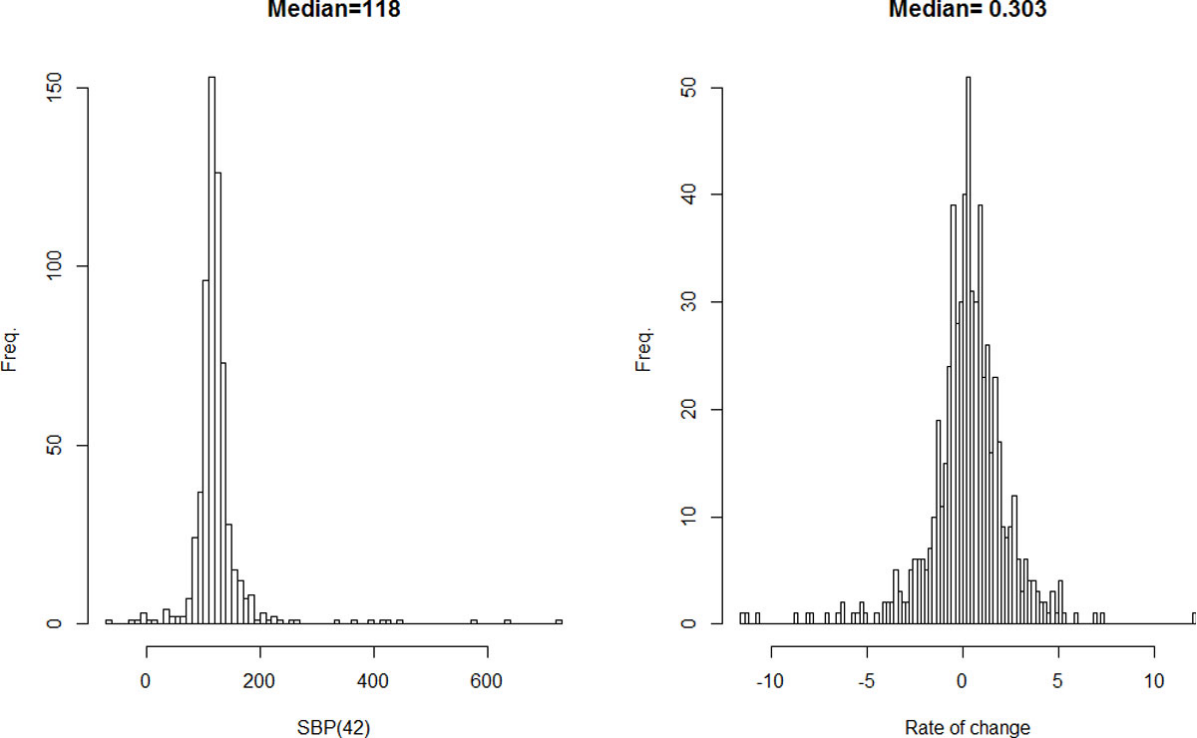
**Histograms for the estimated individual SBP at age 42 years, SBP(42), and rate of change**. Although both distributions are approximately normal, there are sporadic outliers at both ends of each distribution.

We started with regressing SBP values on 2 time-varying variables, smoking and intake of antihypertension medicine, to remove their effects, and then fitted the level 1 models to the age trajectories of SBP measured over time. Figure 1 is a histogram for the estimated SBP at age 42 years (the mean age for all ages at examination), or SBP(42), and the slope for each individual. There are outliers at the far ends in the distributions (Figure 1), defined as those whose values are beyond 3 standard deviations from the mean, that were removed in fitting the level 2 models. The median for SBP(42) is 118 mm Hg. The median for the slope is 0.303 mm Hg per year, which means that for every 10-year increase in age, SBP is expected to increase about 3 mm Hg on average.

**Figure 2:**
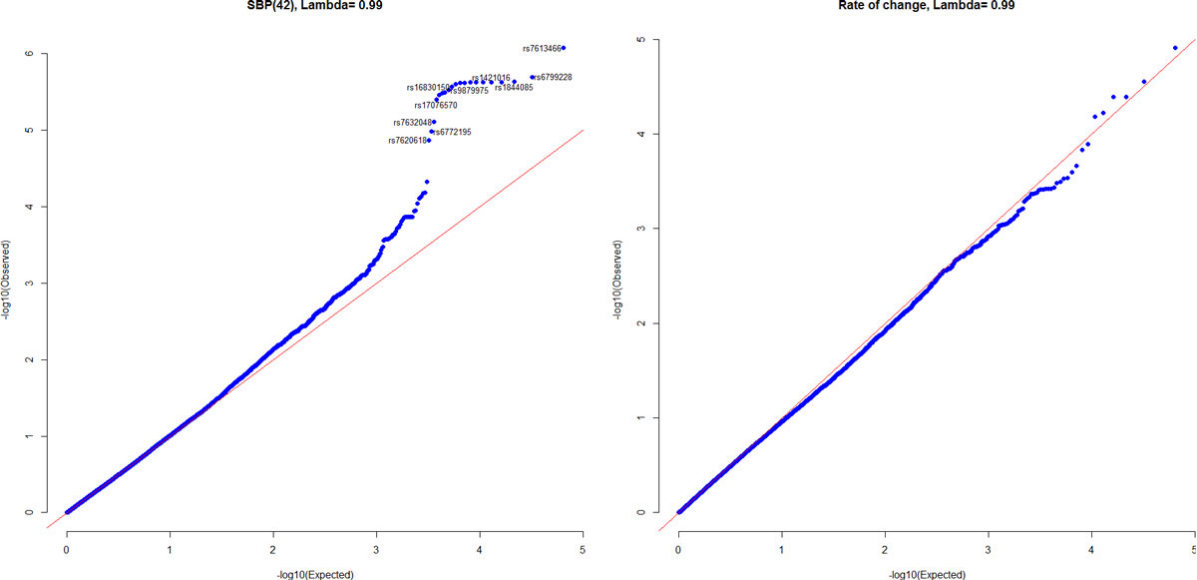
**Q-Q plots for the observed against expected *p *values in association analysis of chromosome 3 data, for SBP(42) (*left panel*) and for rate of change (*right panel*)**.

For each of the 65,519 SNPs genotyped on chromosome 3, we next fitted the level 2 models as described in equations (2) to (6) and included sex as an additional fixed-effect variable. The Q-Q plot is shown in Figure 2 for the *p *values for effects of each SNP on SBP(42) (the left panel) and on rate of change (the right panel). Although no SNP reached genome significance (*p *value <5e-08), some SNPs on chromosome 3 may tend to affect the mean level of SBP (SNP IDs are shown in Figure 2). Meanwhile, our analysis did not reveal any genetic associations that affect the rate of change in SBP for the tested SNPs on chromosome 3. The estimated inflation factors (λ) are 0.99 for both SBP(42) and rate of change, suggesting that our modeling of random effects efficiently removed relatedness in substructures resulting from repeated measurements within individuals and between individuals within pedigrees.

### Simulated phenotype data

The application to original data has shown that the influence of pedigree structure on statistical testing can be nicely controlled by our method. We next use the simulated phenotype data to validate performance of our model and to see whether important genetic variants can be captured for given sample size in the simulation. As with the real data, we focused only on SBP and used regression models to adjust for effects of time-varying variables (smoking and medication) and fitted level 1 model to the residuals. Considering the enormous computational load in performing GWAS for 200 replicates, we limited our analysis on simulated data to all SNPs in the *MAP4 *gene on chromosome 3. The simulation included 14 functional variants (6 with minor allele frequency [MAF] >0.01) accounting for 7.7% of the total variance in SBP. A total of 87 SNPs in the GAW18 GWAS data were mapped to the *MAP4 *gene. We performed level 2 analysis on all 200 replicates, focusing on 62 SNPs with MAF >0.01. Based on the results on simulated replicates, statistical power was calculated as defining type I error rate α = 0.05 and α = 0.05/number of SNPs tested (~0.0008). With a sample size of 849 individuals, the highest power in detecting the effect on mean SBP or SBP(42) was achieved by SNP rs11711953 (MAF = 0.028) with power estimates of 1 and 0.70 for α values of 0.05 and 0.0008. This SNP is the variant in *MAP4 *assigned the highest effect on SBP, accounting for 2.78% of the variance in SBP. Interestingly, we also obtained the highest power for detecting association with rate of change from the same SNP (0.75 for α = 0.05), suggesting that rs11711953 influences both the mean level (negative correlation) and the rate of change over ages (positive correlation) of the simulated SBP. Table 1 displays the top 10 SNPs showing the highest power. As can be seen, the statistical power for each of the SNPs depends on the effect and frequency of the genetic variants in their vicinity and the distance to them. Interestingly, the functional variants in the close vicinity of the top 6 SNPs explain 7.5% of the total variance in SBP, which is nearly the total effect of the *MAP4 *gene specified in the simulation.

**Table 1:** The top 10 SNPs (MAF >0.01) detected with the highest statistical power from simulated data

			SBP(42)		Rate of change		Closest functional variants in simulation			
			
SNP	Position	MAF	Power α = 0.05	Beta mean	Power α = 0.05	Beta mean	Position	MAF	Beta	% Variance
rs11711953	48040283	0.03	1.00	−18.6	0.75	0.59	48042083, 48042084	0.03 0.01	−9.91 −11.01	0.028 0.011
rs1665982	47905079	0.11	0.64	−6.08	0.46	0.25	48042083, 48042084, 47913455	0.03 0.01 0.005	−9.91 −11.01 −8.70	0.028 0.011 0.004
rs319680	47898307	0.15	0.53	−4.60	0.41	0.20	47913455	0.005	−8.70	0.004
rs6763824	47905427	0.14	0.46	−4.47	0.38	0.20	47913455	0.005	−8.70	0.004
rs184388	47939626	0.34	0.40	−3.03	0.33	0.13	47956424	0.38	−2.38	0.014
rs1060407	47958037	0.34	0.39	−3.02	0.33	0.13	47957996, 47957741	0.03 0.002	−7.39 −8.10	0.015 0.003
rs7430879	48038714	0.38	0.39	−2.82	0.39	0.13	48042083, 48042084	0.03 0.01	−9.91 −11.01	0.028 0.011
rs4599334	48066400	0.35	0.37	−2.93	0.29	0.12	48042083, 48042084	0.03 0.01	−9.91 −11.01	0.028 0.011
rs4296617	48068308	0.35	0.37	−2.92	0.28	0.12	48042083, 48042084	0.03 0.01	−9.91 −11.01	0.028 0.011
rs2053767	47999674	0.38	0.37	−2.75	0.38	0.13	48042083, 48042084	0.03 0.01	−9.91 −11.01	0.028 0.011

## Discussion

In longitudinal investigations, researchers are interested in studying interindividual differences in intraindividual changes. Such studies are inherently hierarchical in nature. The nested structure in data includes multiple or repeated observations within an individual, and a collection of multiple individuals in a sample, which can be modeled by HLM. The nested modeling is actually equivalent to the mixed-effects model from a theoretical prospective. By incorporating equations (2) and (3) into equation (1), we obtain

(7) $$Y_{i,j}=\gamma_{00}+\gamma_{10}X_{ij}+\gamma_{01}G_{i}+\gamma_{11}G_{i}X_{ij}+U_{1,i}X_{ij}+U_{0,i}+r_{ij}$$

The combined model has fixed effects for age, genotype and their interaction, and the composite error including random effects for the age trajectory and for the mean SBP level. Unlike the mixed model that corrects phenotype correlation within pedigree, our modeling strategy allows a direct adjustment of kinship correlation in the mean level and the rate of change of SBP. In a combined model, however, this is somewhat more difficult to implement. As shown by the application example, our hierarchical modeling strategy enables integration of kinship matrix to explicitly account for the genetic correlation both in the level and in the rate of change of SBP in general pedigrees using readily available software packages. For comparison, we performed similar association analysis on chromosome 3 but using the linear mixed model with individual rate of change as dependent variable and assuming random effect for each pedigree. The estimated inflation factor becomes 1.07, indicating a moderate degree of sample substructure. This is in contrast with a lambda of 1 from the lmekin model, suggesting that explicitly modeling the kinship correlation structure can help to improve model performances for GWAS in pedigree studies.

Our hierarchical modeling procedure also offers an easy way to detect outliers in longitudinal studies [16], which is easily done by examining the distribution of the estimated individual intercepts and slopes (see Figure 1). For example, in our analysis, we define outliers as any observation with values for the intercept or SBP(42) and for the slope or rate of change beyond 3 standard deviations from the mean. In addition, we also set those with an estimated SBP(42) of less than 60 mm Hg as outliers because it is unreasonable for SBP to be too low to maintain physical function (normal range for SBP: 90 to 140 mm Hg). Because the individual intercept and slope are based on the level 1 model fitted to the repeated measurements on each participant, our multilevel modeling process actually provides summary metrics that can be used for detecting outliers. Note that, in longitudinal studies, the increasing number of waves for data collection can complicate the search for outliers. However, this is an advantage for our way of defining outliers because, with repeated observations, the resulting random slope for each individual can be more stabilized and definition of outliers less affected by occasional extreme point observations.

Finally, although only chromosome 3 data are used as an example, application of the method to genome-wide data is straightforward. The fast fitting of the mixed model with a predefined kinship matrix enables computational feasibility for large GWAS data. With the increasing popularity of GWAS, we hope that the proposed method can be helpful in analyzing data of related individuals using both cross-sectional and longitudinal designs.

## Conclusion

Hierarchical linear modeling of longitudinal pedigree data can handle relatedness in detecting genetic variations that affect the mean level or the rate of change for a phenotype of interest in genetic association analysis.

## Competing interests

The authors declare that they have no competing interests.

## Authors' contributions

TAK, KC, and JBH designed the overall study; QT, AKJ, and JZ conducted statistical analyses. QT, MT, and LC drafted the manuscript. All authors read and approved the final manuscript.

## Declarations

This article has been published as part of *BMC Proceedings *Volume 8 Supplement 1, 2014: Genetic Analysis Workshop 18. The full contents of the supplement are available online at http://www.biomedcentral.com/bmcproc/supplements/8/S1. Publication charges for this supplement were funded by the Texas Biomedical Research Institute.
